# Comprehensive Landscape of HOXA2, HOXA9, and HOXA10 as Potential Biomarkers for Predicting Progression and Prognosis in Prostate Cancer

**DOI:** 10.1155/2022/5740971

**Published:** 2022-03-24

**Authors:** Yan-ping Song, Peng Xian, Hong Luo, Jun-yong Dai, Yu Bai, Yuan Li, Xian-li Tang

**Affiliations:** Chongqing Key Laboratory of Translational Research for Cancer Metastasis and Individualized Treatment, Chongqing University Cancer Hospital, Shapingba District, Chongqing 400030, China

## Abstract

Prostate cancer (PCa) is recognized as a common malignancy in male patients. The homeobox A cluster (HOXA) family members have been confirmed to be implicated in the development of several types of tumors. However, the expression pattern and prognostic values of HOXA genes in PCa have not been investigated. In this study, we analyzed TCGA datasets and identified six HOXA family members which showed a dysregulated expression in PCa specimens compared with nontumor specimens. We also explored the potential mechanisms involved in the dysregulation of HOXA family members in PCa, and the results of Pearson's correlation revealed that most HOXA members were negatively related to the methylation degree. Moreover, we explored the prognostic values of HOXA family members and identified six survival-related HOXA members. Importantly, HOXA2, HOXA9, and HOXA10 were identified as critical PCa-related genes which were abnormally expressed in PCa and associated with clinical outcomes of PCa patients. Then, we explored the association between the above three genes and immune cell infiltration. We observed that the levels of HOXA2, HOXA9, and HOXA10 were associated with the levels of immune infiltration of several kinds of immune cells. Overall, our findings identified the potential values of the HOXA family for outcome prediction in PCa, which might facilitate personalized counselling and treatment in PCa.

## 1. Introduction

Prostate cancer (PCa) is the most commonly seen male reproduction system cancer, which is the 3^rd^ most commonly seen causes of mortality from tumors in male globally [1, 2]. Prostate-specific antigen (PSA) is utilized as a primary biomarker for PCa screening, diagnoses, and prognoses [3]. However, PSA alone as a biomarker still exhibits remarkable restrictions in the diagnosis and prognoses of PCa. Due to insufficient special symptoms of PCa in the early phase, it is easily confused with benign prostatauxe [4, 5]. When evident symptoms occur, the cancers are usually in the middle and advanced phases, even with metastases [6]. Hence, the sufferers usually miss the optimal therapeutic opportunity. Hence, it is of great significance to search for novel biological markers which effectively predict the risk and progression of PCa. Mammals have 39 homeobox A cluster (HOXA) genes arrayed in 4 linearity clusters with 9 to 11 genes each. On the foundation of homology, the genes are divided into 13 paralogous groups [7, 8]. HOXA genes are needed for the normal developmental process of organs, like the central nervous system (CNS), axial bone, limbs, guts, hematogenous and genitourinary tracts, and internal genitalia and externalia [9, 10]. Hence, the aberrant regulation of those genes might induce the progression of malignancies. In recent years, the expression and function of HOXA genes have been reported in several tumors. For instance, HOXA4 expression was remarkably reduced in pulmonary carcinoma, and its overexpression inhibited cellular proliferative, migratory, and invasive abilities by modulating the Wnt signal path [11]. It was reported that silencing lncRNA HOXA10-AS reduced the cellular proliferative ability of oral carcinoma and HOXA10-antisense RNA can be a new prognosis predicting factor [12]. Importantly, several previous studies have reported that some novel prognostic models exhibited a strong ability in predicting the prognosis of PCa patients [13–15]. However, the function of HOXA genes was rarely reported in PCa.

The present research was the first to report the expression profile and prognosis significance of the HOXA family members in PCa patients. Our findings will help in the development of the identification of novel biomarkers for PCa patients.

## 2. Materials and Methods

### 2.1. Workflow

**Figure 1:**
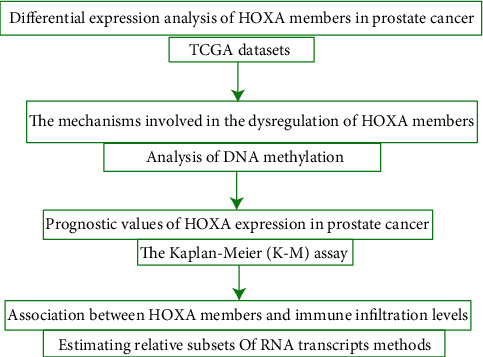
Study flowchart.

We used a combination of methods in several steps to explore the expressing pattern and prognostic values of HOXA family members in PCa and further study the association between HOXA family members and immune cell infiltration (Figure 1).

### 2.2. Data Downloading

The pretreatment information of Level 3 mRNA expressing data was acquired from TCGA database. Clinic specimens associated with PCa and methylated DNA data were chosen. Such dataset involved 499 PCa specimens, 52 normal specimens, and clinic data of those relevant specimens.

### 2.3. Differentially Expressed HOXA Family Members

Data analyses of differentially expressed HOXA genes between PCa and healthy specimens were completed via package limma in R, with liminal values of ∣log2 fold change (FC) | >2 and modified *P* < 0.05. The visualization of the outcomes was realized via the pheatmap package.

### 2.4. Analysis of DNA Methylation of HOXA Family Members

The relationship between the methylation of HOXA family members and their mRNA expression was determined using the Pearson correlation analysis. The annotation of the information on cg spots from Illumina Human Methylation 450K was realized via the annotation document from the Illumina website.

### 2.5. Evaluation of Immune Cell Infiltration

The tumor-infiltrating immune cells (TIICs) in PCa specimens from TCGA cohort and normal prostate specimens from the GTEx database were computed via the CIBERSORT deconvolutional arithmetic. CIBERSORT utilized the white blood cell genetic hallmark matrix (LM22), which involved a series of bar code genetic hallmark matrices of 547 biomarker genes for the quantification of 22 TIICs. The correlation between HOXA family members and TIICs was also estimated using the Pearson correlation analysis.

### 2.6. Statistical Analysis

The entire statistical analysis was completed via R program 3.5.3. The Kaplan-Meier (K-M) assay was finished to study the survival diversities between the high group and the low group. A *P* value < 0.05 was considered statistically significant.

## 3. Results

### 3.1. Expression Status of HOXA Members in PCa Samples

**Figure 2:**
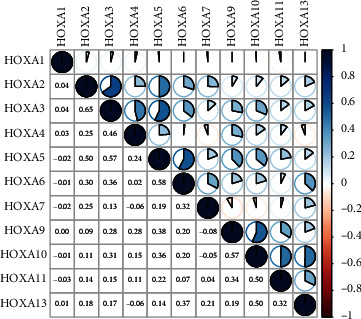
Relationship between HOXA family members.

**Figure 3:**
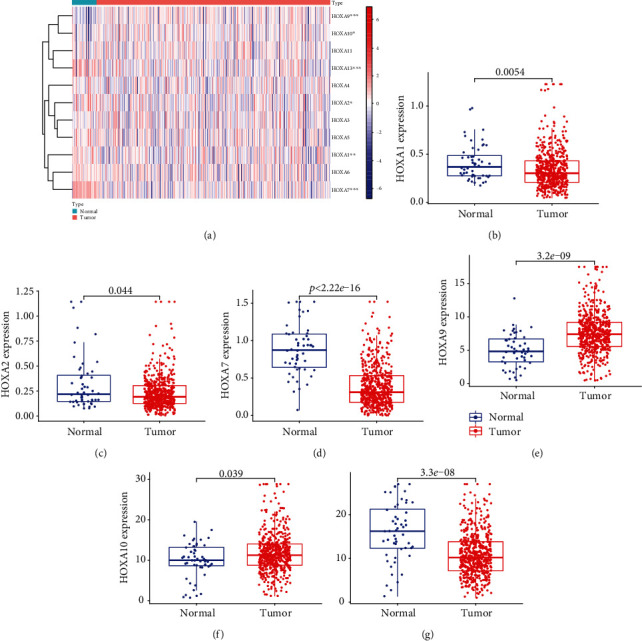
Expression profile of HOXA members in PCa reflected via a heat map (a) and histograms (b–g).

Firstly, the mRNA expressing data on HOXA members (HOXA1-13) from 499 PCa specimens and 52 healthy controls, which derived from TCGA, were acquired via the Perl program. Pearson's correlation of HOXA family genes was computed and utilized to evaluate if those genes were interrelated via the corrplot package. As presented in Figure 2, these genes were remarkably interrelated. Then, the differential expression of these genes was studied via the limma package and visualized via the pheatmap package, as presented by Figure 3(a). Importantly, we observed that the expression of HOXA1, HOXA2, and HOXA7 was distinctly increased in PCa specimens compared with nontumor specimens (Figures 3(b)–3(d)), while the contents of HOXA9 and HOXA10 were distinctly regulated in PCa samples in contrast to nontumor samples (Figures 3(e) and 3(f)). HOXA13 exhibited a decreased expression in PCa tissues (Figure 3(g)).

### 3.2. Association between HOXA Expression and Methylation in PCa

**Figure 4:**
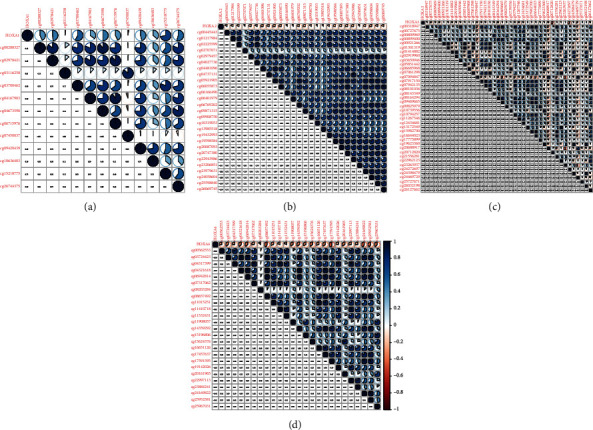
Pearson's correlation between methylation degrees and the expression of (a) HOXA1, (b) HOXA2, (c) HOXA3, and (d) HOXA4.

**Figure 5:**
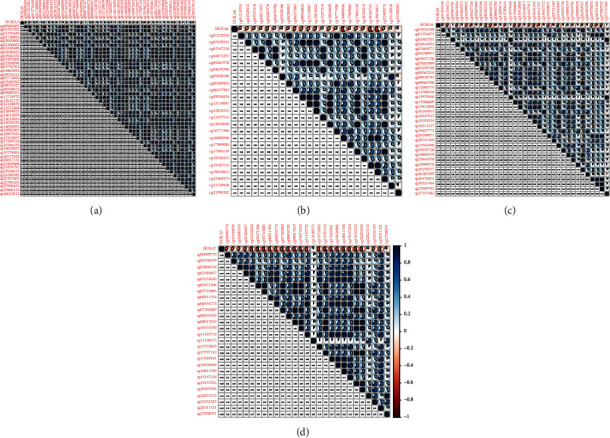
Pearson's correlation between methylation levels and expression of (a) HOXA5, (b) HOXA6, (c) HOXA9, and (d) HOXA7.

Methylated genetic promotor regions affect genetic expression in the development of mankind tumors [16, 17]. Our team further analyzed the association between HOXA expression and methylation in PCa. The results of Pearson's correlation revealed that most HOXA members were negatively related to the methylation degree (Figures 4(a)–4(d) and 5(a)–5(d) and Figures S1A-S1C). Those outcomes revealed the negative association between the expression and methylation degree of HOXA members in PCa.

### 3.3. The Prognosis Value of HOXA Members in PCa

**Figure 6:**
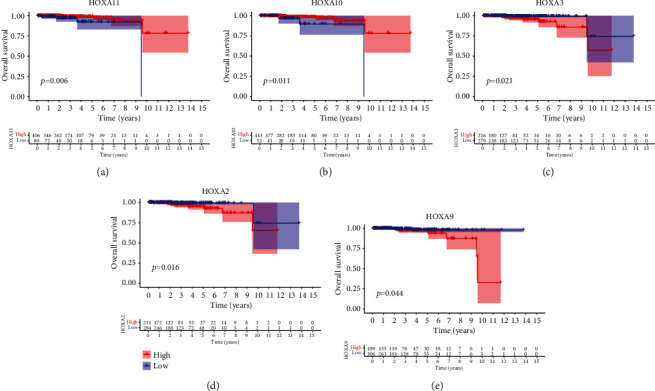
K-M curves for the OS of sufferers in the high and low groups based on the mean expression of (a) HOXA11, (b) HOXA10, (c) HOXA3, (d) HOXA2, and (e) HOXA9 in TCGA cohort.

**Figure 7:**
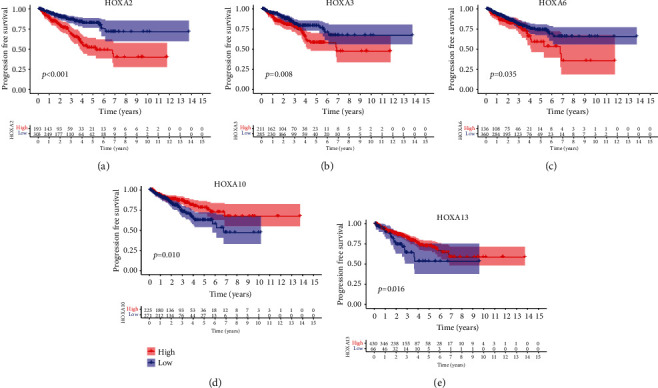
K-M curves for the progression-free survival of sufferers in the high and low groups based on the mean expression of (a) HOXA2, (b) HOXA3, (c) HOXA6, (d) HOXA10, and (e) HOXA13 in TCGA cohort.

For the sake of investigating the clinical value of HOXA members in PCa patients, our team performed Kaplan-Meier methods based on TCGA datasets. Importantly, we observed that high expressions of HOXA11 and HOXA10 were related to a shorter overall survival (OS) of PCa patients (Figures 6(a) and 6(b)), while high expression of HOXA3, HOXA2, and HOXA9 exhibited an opposite result (Figures 6(c)–6(e)). Moreover, we found that sufferers with high expression of HOXA2, HOXA3, and HOXA6 had poor progression-free survival in contrast to those with low expression of HOXA2, HOXA3, and HOXA6 (Figures 7(a)–7(c)), while high expression of HOXA10 and HOXA13 was associated with favorable progression-free survival (Figures 7(d) and 7(e)).

### 3.4. Correlation of HOXA Members with the Proportion of TICs

**Figure 8:**
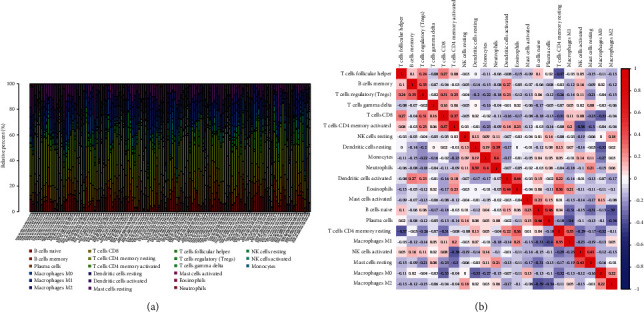
(a) The abundance of 22 infiltrating immunocyte subsets in tumorous and normal biopsies for TCGA cohorts computed via the CIBERSORT approach. (b) Diverse association features amongst 26 immunocyte subsets in PCa cohorts.

**Figure 9:**
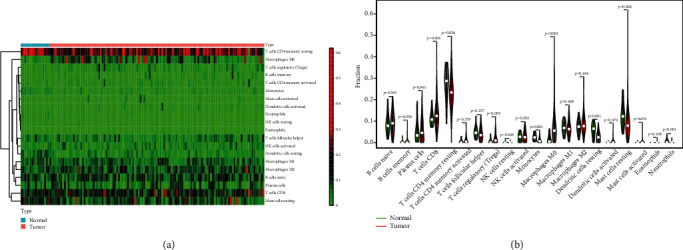
(a, b) Comparison of every kind of immunocyte between PCa and nontumor samples.

**Figure 10:**
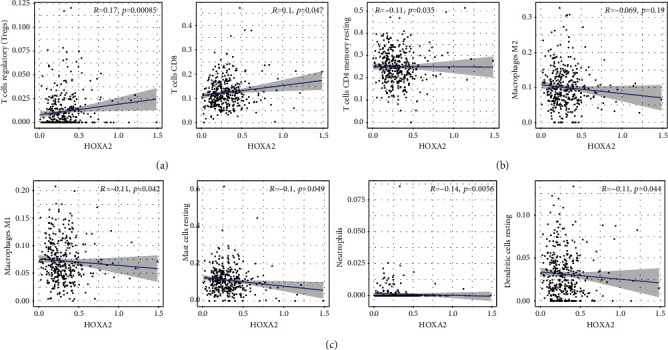
(a–c) Scatter plot presenting the association of 8 types of TIC proportion with the expression of HOXA2.

**Figure 11:**
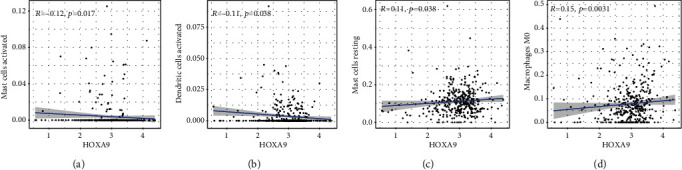
(a–d) Scatter plot presenting the association of 4 types of TIC proportion with the expression of HOXA9.

**Figure 12:**
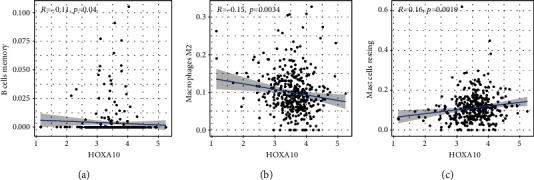
(a–c) Scatter plot presenting the association of 3 types of TIC proportion with the expression of HOXA10.

To verify the association between the expressions of HOXA members and the immunity-related microenvironment, the percentage of cancer-infiltrating immunity subsets was studied via CIBERSORT arithmetic, and 21 types of immunocyte profiles in PCa specimens were established (Figures 8(a) and 8(b)). In contrast to healthy specimens, diverse features of the infiltrating immunocytes in PCa were presented in Figures 9(a) and 9(b). Given that HOXA2, HOXA9, and HOXA10 were dysregulated in PCa and predicted a clinical outcome, we further explored their associations with the level of immune cells. Our team discovered that the HOXA2 content was related to the content of T cells modulatory (Tregs) and T cells CD8 in a positive way (Figure 10(a)), while negatively associated with Macrophages M2, Macrophages M1, mastocytes resting, neutrophils, and dendritic cells (DCs) resting (Figures 10(b) and 10(c)). In addition, we found that the level of HOXA9 was negatively related to the level of mastocytes stimulated and dendritic cells activated (Figures 11(a) and 11(b)), while positively associated with mast cells resting and Macrophages M0 (Figures 11(c) and 11(d)). Moreover, we found that the level of HOXA9 was related to the level of B cells memory and Macrophages M2 in a negative way (Figures 12(a) and 12(b)), while positively associated with the level of mast cells resting (Figure 12(c)).

## 4. Discussion

Carcinoma is still a serious threat to our health, and its prevalence has presented an elevating tendency in recent years [18]. However, the metastatic causal link in tumor sufferers remains elusive, although metastases can forecast poor prognoses. At present, determining new molecule biomarkers is imperative for the sake of estimating tumorous metastases, as those biomarkers are pivotal for tumor therapies and forecasts [19, 20]. Recently, more and more studies have reported that some dysregulated genes were associated with clinical outcomes of tumor patients, including PCa [21, 22]. In this study, we focused on HOXA genes.

In this study, we identified six HOXA genes which exhibited a dysregulated level in PCa via analyzing TCGA datasets, including HOXA1, HOXA2, HOXA7, HOXA9, HOXA10, and HOXA13. Previously, Malek et al. reported that suppressing HOXA9 pharmacologically avoided TWIST1-triggered invasive PCa cells in vitro and metastases in vivo, indicating that HOXA9 served as a tumor promotor in PCa [23]. Dong and his group showed that HOXA13 expression was distinctly increased in PCa samples and forecasted inferior prognostic results of PCa sufferers. Functionally, they found that forced expressions of HOXA13 evidently facilitated oncocyte growth, metastasis, and aggression, but it suppressed the programmed cell death of oncocytes [24]. However, the function of other HOXA genes was not reported in PCa. To explore the mechanisms involved in the abnormal expression of HOXA genes in PCa, we analyzed the association between HOXA expressions and the methylation degree of cg spots in the promotor regions in PCa, finding that most differentially expressed HOXA members were influenced by the methylation degree, which was consistent with previous findings in acute myeloid leukemia patients and laryngeal squamous cell cancer patients.

For the purpose of investigating the clinic value of HOXA genes in PCa, we downloaded survival data using TCGA datasets. We observed that the expression of HOXA1, HOXA2, HOXA3, HOXA10, and HOXA9 was related to the OS of PCa sufferers. Moreover, the expressions of HOXA2, HOXA3, HOXA6, HOXA10, and HOXA13 were associated with progression-free survival of PCa patients. Previously, several studies have also reported the prognostic value of HOXA genes in some cancers, like cervical carcinoma and acute myeloid leukemia [25–27]. Our findings, together with previous studies, suggested the probability of HOXA genes used as novel biomarkers for PCa patients.

As progress in molecule-level researches, tumor-infiltrating immune cells can facilitate and/or modulate cancer development via the cell types and their mutual effects [28]. In recent years, in genitourinary cancers, there has been remarkable progress in terms of immunocyte infiltrates, whereas their effects on tumorigenesis and prognoses are still elusive [29, 30]. In this study, our team chose HOXA2, HOXA9, and HOXA10 to analyze their association with immune infiltrates. We observed that the expression of HOXA2 was associated with DC resting, M1, M2, mastocyte resting, neutrophilic cells, T cells CD4 memory resting, T cells CD8, and T cells regulatory (Tregs), and the expression of HOXA9 was associated with DCs stimulated, Macrophages M0, mastocytes stimulated, and mastocyte resting. Our finding suggested the involvement of HOXA9 and HOXA2 with immune infiltrates.

Nevertheless, certain deficiencies of our research ought to be disclosed. Firstly, the race in TCGA database was predominantly White and Black people, and the extrapolation of the discoveries to other races had to be corroborated. Secondly, the specimen size of sufferers was inadequate. In addition, this research merely highlighted biological information analysis. In vivo and in vitro assays were needed to substantiate the outcomes of our research.

## 5. Conclusion

The present study provides fresh enlightenment pertaining to the favorable effect of HOXA family members on the prognoses and development of PCa, paving a way for novel HOXA-targeting treatment regimens for PCa.

## Data Availability

The data used to support the findings of this study are available from the corresponding author upon request.

## Supplementary Materials

Supplementary Materials Figure S1: Pearson's correlation between methylation degrees and the expression of (A) HOXA10, (B) HOXA11, and (C) HOXA13.
