# Supplementary figures and images for: Comprehensive Landscape of HOXA2, HOXA9, and HOXA10 as Potential Biomarkers for Predicting Progression and Prognosis in Prostate Cancer
Source: J Immunol Res. 2022 Mar 24;2022:5740971. doi: 10.1155/2022/5740971 (PMC8970952; doi:10.1155/2022/5740971)

A

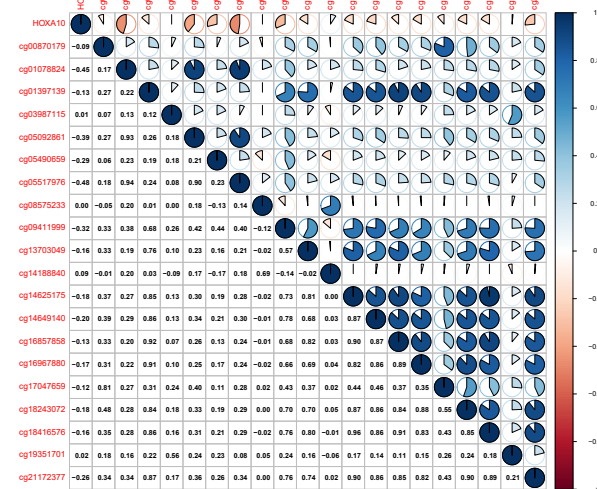

B

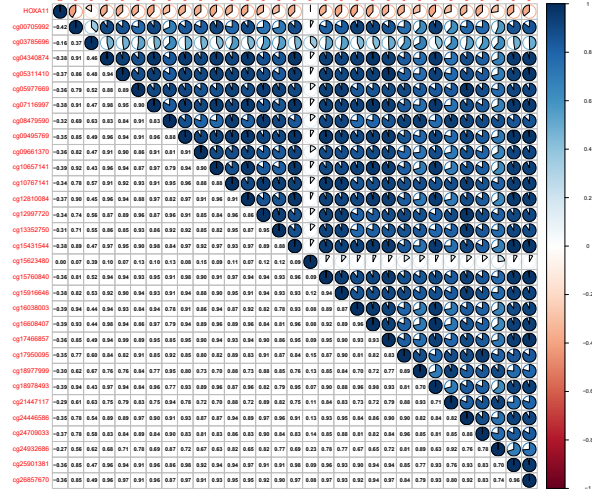

C

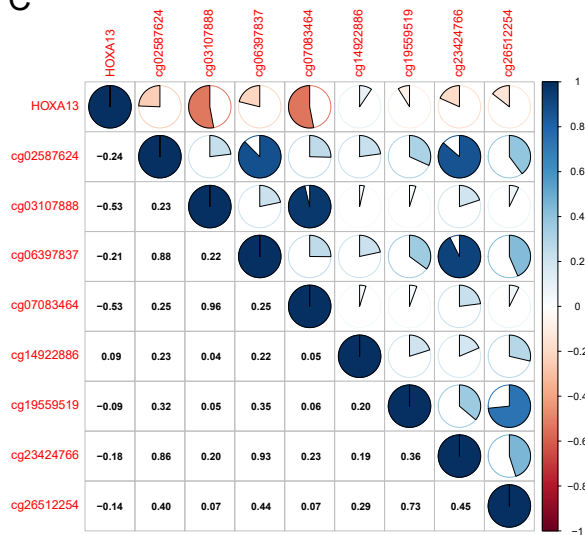

Supplement: Supplementary Materials — Figure S1: Pearson's correlation between methylation degrees and the expression of (A) HOXA10, (B) HOXA11, and (C) HOXA13. [file 5740971.f1.pdf]
